# Mapping Biological Risks Related to Necropsy Activities: Old Concerns and Novel Issues for the Safety of Health Professionals

**DOI:** 10.3390/ijerph182211947

**Published:** 2021-11-13

**Authors:** Paola Tomao, Raffaele La Russa, Alessandra Oliva, Massimiliano De Angelis, Antonella Mansi, Emilia Paba, Anna Maria Marcelloni, Alessandra Chiominto, Martina Padovano, Aniello Maiese, Matteo Scopetti, Paola Frati, Vittorio Fineschi

**Affiliations:** 1 Department of Occupational and Environmental Medicine, Epidemiology and Hygiene, Italian Workers’ Compensation Authority (INAIL), 00078 Rome, Italy; p.tomao@inail.it (P.T.); a.mansi@inail.it (A.M.); e.paba@inail.it (E.P.); a.marcelloni@inail.it (A.M.M.); a.chiominto@inail.it (A.C.); 2 Department of Clinical and Experimental Medicine, University of Foggia, 71122 Foggia, Italy; raffaele.larussa@unifg.it; 3 IRCSS Neuromed Mediterranean Neurological Institute, Via Atinense 18, 86077 Pozzilli, Italy; alessandra.oliva@uniroma1.it (A.O.); paola.frati@uniroma1.it (P.F.); 4 Department of Public Health and Infectious Diseases, Sapienza University of Rome, 00185 Rome, Italy; massimiliano.deangelis@uniroma1.it; 5 Department of Anatomical, Histological, Forensic and Orthopaedic Science, Sapienza University of Rome, Viale Regina Elena 336, 00185 Rome, Italy; martina.padovano@uniroma1.it (M.P.); matteo.scopetti@uniroma1.it (M.S.); 6 Section of Legal Medicine, Department of Surgical Pathology, Medical, Molecular and Critical Area, University of Pisa, 56126 Pisa, Italy; aniello.maiese@unipi.it

**Keywords:** necropsy activity, biological risk, microbiology, SARS-CoV-2, risk management, occupational risk

## Abstract

Nowadays only a few studies on biological and environmental risk among healthcare workers are available in literature. The present study aims to assess the health operator’s risk of contact with microorganisms during necropsy activities, to evaluate the efficiency of current protections, to identify possible new sources of contact, and to point out possible preventive measures. In addition, considering the current pandemic scenario, the risk of transmission of SARS-CoV-2 infection in the dissection room is assessed. The objectives were pursued through two distinct monitoring campaigns carried out in different periods through sampling performed both on the corpses and at the environmental level.

## 1. Introduction

Biological risk refers to the probability of being exposed to one or more microorganisms that cause a life-threatening disease, resulting in death or permanent disability. Such a risk depends on several microorganisms identified by the World Health Organization (WHO) in Appendix UN2814 [1].

In all healthcare activities that expose healthcare workers to blood or other biological fluids through organs or tissues, there is a real risk for operators of contracting an infectious disease. The risk of professionally contracting infectious diseases during necropsy has been known and documented in the literature for a long time owing to the frequency of accidents (stings, cuts, etc.) that can occur during the various activities. The most documented infections in this sector are attributable to the transmission of HIV virus [2], hepatitis viruses (HBV and HCV) [3], Mycobacterium tuberculosis [4,5,6], agents responsible for hemorrhagic fevers and prions [7,8].

In this context, conducting risk analysis is needed. In fact, risk analysis allows us to achieve risk control, decrease the frequency of accidents, reduce their severity, and minimize the risk of biosafety laboratory operations with minimal cost.

The operators of necropsy services can come into contact with pathogens by both direct and indirect exposure [9]. Direct exposure can occur by inoculation from accidental punctures or wounds from tools or sharp objects contaminated with blood or other biological fluids; direct transmission can also occur by mucocutaneous route following contact with splashes of biological materials. Indirect exposure during autopsy can occur by inhalation of aerosol particles with a diameter of less than 5 µm produced during the opening of the thoracic cage and abdominal cavity, the cutting of bones, or following the section of the organs; these particles can, under certain conditions, rapidly spread into the surrounding environment and remain in the air for different amounts of time, contaminating people, surfaces, and equipment.

Risk management during necropsy activities includes an accurate identification and assessment of biological hazards, which presupposes the risk assessment of the variables connected to the operator (identification of the maneuvers at risk) and of the variables connected to the biological agent (possible reduction of the vitality of the pathogens in the presence of transformative cadaveric phenomena, different survival times of each biological agent in the external environment, etc.). Indeed, the biological risk is also attributable to the high prevalence of undiagnosed parenteral communicable diseases among the general population [10,11,12] and the high level of contagion in the health professions [13].

At present, there are scarce and not recent data in the literature regarding the possibility of transmission of pathogens from the dead body to the health worker.

The activities in the autopsy room are diversified and the information relating to the specific tasks, as well as the prevention and protection measures adopted, are useful for the correct management of biological risks. In fact, these data can provide indications for the identification of operating methods that could entail a greater risk of infection and suggest strategies to limit the spread of pathogenic microorganisms in the workplace and to exposed personnel.

In compliance with the precautionary principles, all deceased subjects must be considered potentially infected and can represent a risk to the health of anyone who comes into direct contact (family member or operator), the rationale underlying the current research is to identify the sources of occupational risk in the performance of the various necropsy activities, as well as to identify the criticalities of the operating procedures considered at greatest risk of transmission from occupational exposure.

The present study aims to assess microorganisms health operators could come into contact with during necropsy activities, to evaluate whether current protections are suitable, to identify possible new sources of contact, and to point out possible preventive measures [14,15,16]. In addition, the study pursues the objective of searching for pathogens on samples taken from the corpse during autopsy; then, considering the current pandemic scenario, the risk of transmission of SARS-CoV-2 infection in the dissection room is assessed, among others. The objectives were pursued through two distinct monitoring campaigns carried out in different periods through sampling performed both on the corpses and at the environmental level. Compared to the previous one, the monitoring campaign for the search for SARS-CoV-2 included only the molecular analysis of the swabs carried out on the corpse; the methodological change was made owing to the need to limit the number of operators present in the sector room during autopsies and the priority given to sanitation procedures at the end of the investigations.

## 2. Materials and Methods

The study was conducted in the dissection hall and the annexed rooms of the Legal Medicine Unit of the Umberto I General Hospital in Rome. The monitoring and sampling activities were carried out from September to December 2016 as regards the cadaveric and environmental investigations on the most widespread and well-known microorganisms; a further sampling campaign aimed at searching for SARS-CoV-2 on cadaveric samples was carried out from March 2020 to May 2021. Concerning the selection criteria and the sample size, all the activities carried out in the period under investigation were monitored in the absence of exclusive criteria regarding the type of assessment, post-mortem interval, gender, age, and post-mortem diagnosis; similarly, regarding monitoring for SARS-CoV-2, all the corpses accepted in the study period were examined. The sampling involved all the instrumentation supplied to the morgue, the dissection hall, and the annexed laboratories (Table 1).

The room where the autopsies were performed presented characteristics compatible with the biosafety level 3 (BSL3); in particular, the room was equipped with negative pressure and 13 air changes per hour with the expulsion of the air to the outside through HEPA filters.

Considering the different risk categories, the homogeneous groups of workers involved in necropsy activities were exposed to the risk of injuries from the handling of sharp material (saws, scalpels, surgical forceps, etc.) and contact between the mucous membranes or the injured skin and objects and surfaces contaminated with corpse material.

A critical review of the autopsy maneuvers was carried out in order to characterize the activities with the greatest risk of damage to the psychophysical integrity of the worker. After the analysis of the processes, homogeneous groups of subjects at risk were identified in the activities of transferring the bodies, inserting and removing the bodies in the refrigeration units, washing of stretchers and dishes, hygienic and conservative treatments, autopsy, storage and treatment of samples. The operators actively collaborated to make monitoring possible through the application of detection instrumentation (such as personal aspirators) with the execution of standardized and regularly recorded maneuvers at the moment of execution.

### 2.1. Questionnaires

Clinical-anamnestic questionnaires were elaborated according to a validated scheme, structured in an anonymous format, and filled in by the operators only after having signed the informed consent. The questions comprehended age and sex, professional qualification and job seniority, use of personal protective equipment (PPE), adoption of suitable behavior and specific prevention measures (vaccinations, hand washing, sanitation of the workplace). The data from the questionnaires were entered in an Excel sheet to obtain a database for statistical analysis; precisely, a descriptive analysis of categorical variables with the description of the frequencies in absolute terms and in percentage was performed.

### 2.2. Cadaveric Samples

Cadaver sampling was performed through the collection and storage in sterile containers of respiratory secretions (bronchial swab), lung tissue samples, and blood (intraventricular puncture). After collection, the samples were stored in the refrigerator at +4 °C until subsequent analyses. The plasma and serum samples were stored in the refrigerator at −20 °C until further analysis. Samples of respiratory secretions, lung tissue and blood were subjected to bacteriological tests consisting of:

▪ a search for common germs and fungi; each sample was seeded on general (blood Agar) and specific (Mac Conkey, Sabouraud, Mannitol Salt Agar) solid media and incubated for 24–48 h at 37 °C; the quantification of microorganisms was expressed in Colony Forming Units (CFU)/mL;
▪ a search for alcohol-acid fast bacilli (direct and cultural examination); each sample, after an adequate decontamination and fluidization procedure, was subjected to direct examination with Ziehl-Neelsen staining and subsequent incubation for 40 days at 37 °C on a specific medium for mycobacteria (Lowenstein-Jensen).

The plasma and serum samples were subjected to virological tests (Siemens Healthineers) for the detection of HBsAg [17], HCV-Ab [18], and HIV-Ab [19].

### 2.3. Environmental Samples

**Figure 1:**
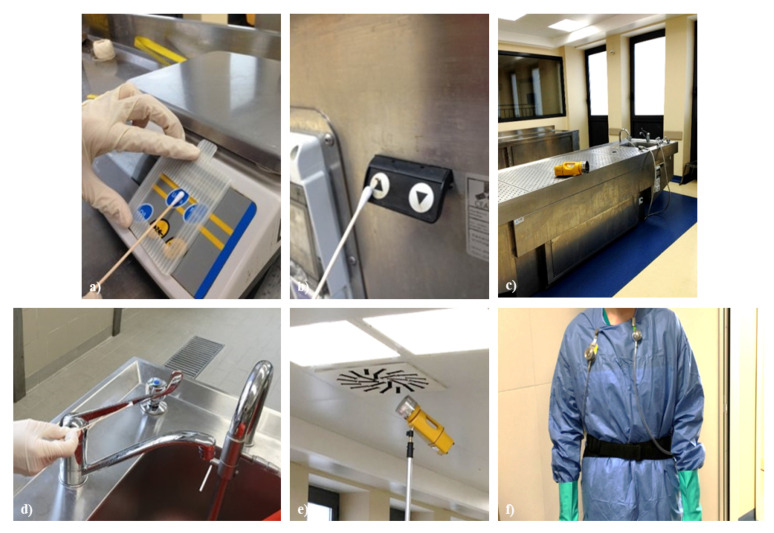
Sampling sites: (**a**) scale keyboard; (**b**) autopsy table button; (**c**) autopsy table; (**d**) tap mixer; (**e**) air intake grids; (**f**) personal sampling by IOM sampler.

Environmental microbiological monitoring was carried out both in an inactive autopsy room and during autopsies. As far as bioaerosol was concerned, the sampling protocol envisaged the use of portable samplers with active aspiration (Surface-Air-System, SAS) in which the air, at a constant speed and for a defined time, was conveyed directly onto Petri plates. These contained generic culture media for total bacterial and fungal counts (Tryptone Soy Agar-TSA, Malt Extract Agar-MEA) and selective media (Mannitol Sal Agar MSA, Mac Conkey Agar-MC3, Slanetz Agar-SA) for the isolation and identification of coagulase positive staphylococci, Gram-negative bacteria, and fecal enterococci. Air samplers, placed near the sampling site (Figure 1), were active during the opening of the cranial, thoracic, and abdominal cavities. During activities, the bioaerosol inhalable fraction was also taken (particle cutting up to 100 µm: particles involving the upper respiratory tract) aimed at assessing the workers’ personal exposure to bacterial endotoxins, integral components of Gram-negative bacteria whose inhalation can have serious health effects. TSA plates were incubated at the temperature of 25 and 36 ± 1 °C for 24–48 h, MC3 and MSA plates at the temperature of 36 ± 1 °C for 24–48 h, those containing MEA at the temperature of 25 and 36 ± 1 °C for at least 5 days up to a maximum of 2 weeks. Each plate was inspected daily, noting the number and characteristics of the individual colonies that had developed. For bacterial endotoxins sampling, the method envisaged the use of suction pumps, set a flow rate of 2 L/min, connected to particle fractionators (IOM sampler, SKC) commonly used for inhalable dust sampling [20], equipped with fiber glass filters (GF/A, diameter 25 mm, 1.6 μm porosity). These devices were worn by the operators for the entire duration of the work shift (Figure 1). The membranes housed inside the particle fractionators were subjected to extraction procedures in pyrogen-free water and Tween 20; the supernatant was analyzed by Kinetic-QCL LAL assay (Lonza Walkersville, MD, USA) for the quantification of bacterial endotoxins, and the results were expressed in EU/m^3^.

Bacterial isolates were identified by biochemical methods (Bio-Mérieux, Marcy l’Etoile, France). For bacterial quantification, tenfold dilutions of the samples were then plated on Muller-Hinton Agar and the number of CFUs determined. Further, multi-drug resistance was defined [21]. Fungi (molds and yeasts) were also identified on the basis of macroscopic and microscopic characteristics. The tests on the surfaces were carried out in an inactive and sanitized room (Figure 1) in order to assess the environmental hygiene level, the effectiveness of the cleaning and/or decontamination procedures and interventions implemented. For this purpose, different sampling methods were used to evaluate both microbiological (Rodac Weigh and swabs) and biological (ATP bioluminescence) contamination from residues of organic material, biological fluids, etc. As regards the surfaces, the plates containing the culture media previously housed in the Rodac Weight were incubated and then subjected to counting and biochemical identification as described above; the results were expressed in CFU/cm^2^. The swabs, after sampling, were placed in phosphate-buffered saline (PBS), subjected to serial dilutions, and seeded on generic and selective culture media. The ATP was quantified in real time with a bioluminometer (Hygiena SystemSURE Luminometer) and the results expressed in RLU/cm^2^ [22], being the amount of light emitted during the reaction directly proportional to the amount of ATP present in the sample collected from the surface.

### 2.4. Research of SARS-CoV-2

In the context of the COVID-19 pandemic and in accordance with current evidence [23], pre-autopsy swabs were performed in the search for SARS-CoV-2. Swabs of the upper respiratory tract (nasopharynx and oropharynx) were taken before the autopsy, whereas swabs of the lower respiratory tract (trachea and primary bronchi) were taken during the autopsy. Immediately after collection, the samples were placed in sterile tubes containing 2 mL of transport medium for subsequent storage in the refrigerator at 2–8 °C before processing. The viral titer in each specimen was estimated using real-time reverse transcription polymerase chain reaction (RT-PCR). Post-mortem swabs were processed using the reagent system for SARS-CoV-2 RT-PCR (RealStar^®^, Altona Diagnostics, Germany). The reagent system was based on real-time PCR technology for qualitative detection and differentiation of lineage B betacoronavirus specific RNA (B-βCoV). Real-time RT-PCR technology enabled the conversion of RNA to complementary DNA through the reverse transcriptase reaction, the amplification of target sequences through the polymerase chain reaction, and the detection of amplified DNA through specific probes labeled with a fluorophore and a quencher. All reagents and samples were completely thawed, mixed (20 µL of reagent and 10 µL of sample or control by pipetting or gentle sweeping on a shaker), and centrifuged for 30 s at approximately 3000 rpm prior to use. The set reaction volume was 30 µL, the ramp rate was predefined, and the passive reference was ROX^TM^. The thermal profile and the acquisition of the dyes were differently set for the reverse transcription (maintenance phase, one repeat of the cycle, no acquisition, temperature of 55 °C, duration of 20 min), denaturation (maintenance phase, one repeat of the cycle, no acquisition, temperature of 95 °C, duration of 2 min) and the amplification (cycling phase, 45 repetitions of the cycle; 15 s at 95 °C without acquisition, 45 s at 55 °C with the acquisition of dyes and 15 s at 72 °C without acquisition). The limit of detection of the RT-PCR was 2000 copies of viral RNA/mL. RNA was quantitatively assessed to distinguish RNA of βCoV lineage B (B-βCoV) from that of SARS-CoV-2. For this, structural E-genes, specific for B-βCoV, and S-genes, specific for SARS-CoV-2, were amplified using RT-PCR, and the cycle threshold (Ct) values were used for analysis. The qualitative analysis applied is presented below (Table 2).

## 3. Results

### 3.1. Questionnaires

**Figure 2:**
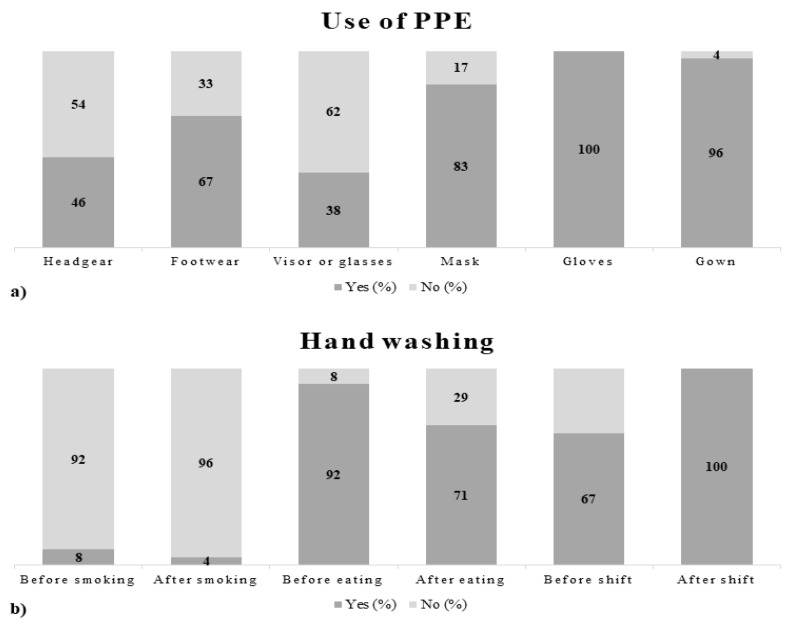
(**a**) Data on use of personal protective equipment (PPE); (**b**) data on hand washing performance.

**Figure 3:**
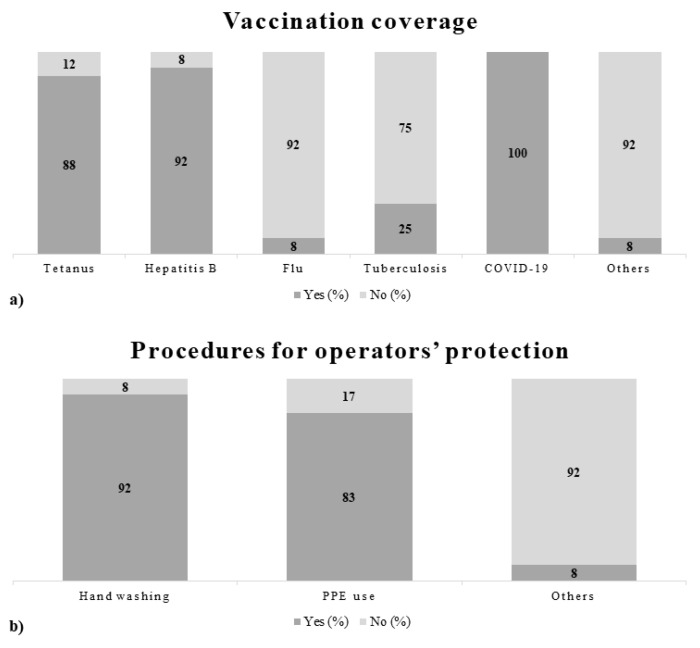
(**a**) Data on vaccination coverage; (**b**) data on the presence of written procedures for workers’ protection.

**Figure 4:**
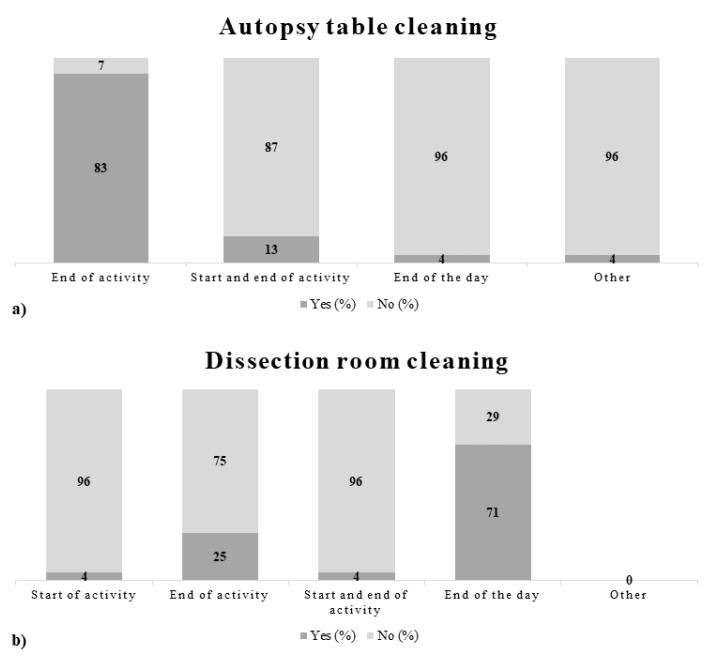
(**a**) Autopsy table cleaning; (**b**) Dissection room cleaning.

The descriptive analysis of data that emerged from the questionnaires made it possible to obtain an overview of the main activities of enlisted subjects and identify the risk profiles related to specific tasks. The specific tasks to be surveyed consisted of performing autopsies, carrying out laboratory tasks, and transporting bodies. To provide a detailed picture of the results, some figures describing behaviors, as well as prevention and protection measures adopted by the working population enrolled in the study are presented below (Figure 2, Figure 3 and Figure 4).

Administration of the questionnaire during the SARS-CoV-2 pandemic period did not produce useful results because of poor staff compliance (only two units).

### 3.2. Cadaveric Samples

The collection of fluids and tissues from corpses involved 20 subjects (18 males and 2 females) with a mean age of 43 years who died from infectious causes (1; 5%) and non-infectious causes (19; 95%). The mean time from death to specimen processing was 3 days (range 1–6).

The search for pathogens on autopsy samples showed a negative direct and cultural examination for M. tuberculosis in all subjects. The search for common microorganisms highlighted the presence of pathogens on blood and lung tissue samples in a fair number of cases (Table 3).

Virological analyses carried out on plasma and serum collected from 8 bodies showed positivity for HBsAg and HIV-Ab, both with low titer, in one of the subjects; high titer HCV-Ab was detected in another of the study subjects.

### 3.3. Environmental Samples

Air microbiological monitoring in inactive, sanitized, and unmanned room showed the presence of total bacterial concentrations between 0 and 30 CFU/m^3^, with the finding of the highest concentrations near the exhaust air intake grids. Concerning the air qualitative aspect, non-pathogenic saprophytic microorganisms belonging to the genera *Staphylococcus* (*S. epidermidis* and *S. haemolyticus*), *Micrococcus* spp., and *Bacillus* spp. were identified. No mycotic agents were found.

As regards the sampling on environmental surfaces in the inactive and sanitized autopsy room, the mean value (CFU/cm^2^) of detected microorganisms (bacteria and fungi) was 180 CFU/cm^2^ (ranging from 0 to 300 at an incubation temperature of 25 °C) and 122 CFU/cm^2^ (ranging from 0 to 525 at an incubation temperature of 37 °C).

Some pathogenic bacterial species as *Staphylococcus aureus*, *Escherichia coli*, *Enterobacter cloacae*, *Enterococcus faecalis*, and *Enterococcus faecium* were detected on specific sites such as the autopsy table button, tap mixer, and scale keyboard. ATP results higher than 100 RLU/cm^2^ confirmed that on some of these surfaces (mainly the push-button panels, keyboards, and electrical socket covers), there was a high level of biological and microbiological contamination [24].

Air samplings carried out during the autopsy activity showed that the bacterial and fungal concentrations in the bioaerosol sampled near the autopsy table were between 186 and 371 CFU/m^3^ (25 °C) and between 119 and 338 CFU/m^3^ (37 °C). Regarding the qualitative aspect, colonies of *Staphylococcus aureus* as well as other microorganisms belonging to Risk Group 2 [25] were isolated from air samples during the skull opening; specifically, colonies of *Klebsiella oxytoca*, *Escherichia coli*, *Enterobacter cloacae* and *Enterococcus faecalis* were detected following the opening of the thoracic and abdominal cavities (Table 4). With regard to investigations on fungal contamination, only microorganisms belonging to Risk Group 1 were identified. The measurements carried out during autopsies on exhumed corpses revealed mean values of airborne bacterial and fungal concentrations ranging between 133 and 457 CFU/m^3^ (25 °C) and between 178 and 445 CFU/m^3^ (37 °C); as regards the qualitative aspect, mainly saprophytes and colonies of *Enterococcus faecalis* (Risk Group 2) were isolated only at the time of opening the thoracic cavity; with regard to fungal contamination, only microorganisms belonging to Risk Group 1 were isolated.

Sampling on operators during the autopsy produced different results based on the state of conservation of the corpse. The concentrations of airborne endotoxins detected in the inhalable fraction of bioaerosol during the autopsies performed on corpses in a good state of conservation (mean value 1.13 EU/m^3^; range 0.22–3.85 EU/m^3^) were below the occupational exposure limits proposed in the literature [26]. During the activities carried out on exhumed corpses a mean value of 37.6 EU/m^3^ was observed; however, a concentration of 95 EU/m^3^ exceeding the recommended occupational exposure limit of 90 EU/m^3^ proposed by DECOS was measured in a single air sample.

### 3.4. Research of SARS-CoV-2

Regarding the detection of SARS-CoV-2 by RT-PCR on samples of the upper and lower respiratory tract, 205 cases were analyzed. There were 35 (17%) positive cases including 23 males (65.7%) and 12 females (34.3%); the mean age of the positive subjects was 68 years (range 36–97). The interval between death and swabs was on average 3.4 days (range 1–30); in 15 cases (42.8%) the positivity was found more than 10 days after death.

## 4. Discussion

The management of all risks related to necropsy activities must necessarily include the accurate identification and assessment of all hazards and exposure patterns and the subsequent adoption of precautions and protections to be taken by all workers involved in such complex activities. The first phase of this process is represented by risk assessment, which consists in identifying and describing hazardous activities. The next phase is called risk analysis (Table 5), and it deals with the identification of all risk factors in relation to the individual activities with the aim of putting in place suitable measures to reduce the risk itself. It represents the method to understand the nature of risk, to provide information support for risk assessment and determination of the most appropriate risk management strategy and method. It allows measuring the impact or loss caused by a particular phenomenon and provides a basis for risk management. In the present study, risk analysis was performed focusing on the specific manual skills and operational maneuvers performed during the activity. During the external inspection, operators are exposed to biological risk as a result of possible contact with infected skin, organic material (feces, vomit, etc.) or biological fluids (blood, secretions, etc.) leaking from natural orifices, from ulcerations or injuries [27]. Similarly, the mobilization of the corpse and the compression of the rib cage can cause the projection of droplets with consequent exposure to biological risk of any operators not adequately protected. The body dissection itself entails risks of exposure because it requires the use of sharp and pointed instruments, places the sector doctor (and whoever assists them) in contact with the viscera and biological fluids, and can determine the formation of aerosols capable of carrying infectious agents; for this last reason, it is essential to activate the suction system of the sector table during the autopsy in order to reduce the risk [28]. During the execution of both hospital and judicial autopsies, in addition to the intrinsic risks related to the improper use of potentially harmful tools, additional risks were identified that can manifest in particular situations and that expose the operator to the transmission of organic agents:

▪ the opening of the cranial cavity and the extraction of the brain are particularly risky because of the release of bone dust at risk of inhalation, the possibility of injury by bone fragments with sharp edges (fracture lesions or section surfaces);
▪ the opening of the thoracic cavity can be risky owing to the possible presence of metal sutures, fracture lesions that are not easily visible;
▪ the opening of the abdominal cavity with extraction of the organs can involve risks from the possible presence of foreign bodies of a medical nature or of other origin (metal fragments, splinters, retained projectiles, etc.) as well as the possible presence of bone stumps from any fracture, especially in the pelvis.

Peculiarities were also identified that characterize the forensic autopsy activity compared to the anatomopathological one:

▪ the anatomopathological activity consists of cases mostly studied clinically;
▪ the forensic activity involves exposure to different and additional risks (contaminated corpses, foreign bodies, etc.).

The monitoring campaigns demonstrated the substantial adequacy of the environment with respect to the internationally recommended values for the various parameters under investigation. In particular, the environmental microbiological monitoring carried out in the autopsy room showed an overall good quality of the environmental microbiological parameters.

Considering the many innovative aspects relating to the identification of possible risk factors during necropsy activities and subsequent development and application of the prevention measures developed, the study indicated the need for an adaptation of the procedures, keeping the risk estimate separate from the assessment. The need to implement preventive measures was also evidenced by the persistence of bacteria in cadaveric liquids. A similar finding was also confirmed by the evidence available in the literature which showed a tuberculous infection rate of 10% in forensic pathologists versus 4% in pulmonology specialists, as well as a vitality of Mycobacterium tuberculosis in biological samples of cadavers 8 days after death and in tissues fixed in formalin 45 days after sampling [29]. Nevertheless, the risk of parenteral transmission of pathogens such as HIV, HBV, HCV, and HTLV-1 was significant in relation to the use of sharp and pointed instruments.

### 4.1. Questionnaires

The data obtained from the administration of the questionnaires generally documented a good adhesion of the working population to technical, organizational, procedural, and hygienic measures, indicating an awareness of the operators toward the risk of exposure to biological agents. The results analyzed were however preliminary, therefore it will be necessary to recruit additional staff and carry out further analyses to increase the significance of the data obtained.

### 4.2. Cadaveric Samples

Regarding the search for pathogens on autopsy material and the analysis of the potential risk of transmission to operators, it should be noted that the data, although referring to a small sample, showed how the method used can highlight and quantify the various pathogens on autopsy blood and tissue samples, even days after death.

Samples examined and evaluated for the presence of alcohol-acid-resistant bacilli demonstrated the absence of M. tuberculosis in all subjects. On the contrary, the positivity of some samples for HIV, HBV, and HCV represented an element to be taken into consideration during necropsy and judicial inspection because of the potential transmissibility of these pathogens.

However, preventive measures are also needed to address the risk of microorganisms persistence in body fluids.

### 4.3. Environmental Samples

The measurements carried out in an inactive and sanitized room showed a good microbiological quality of the air introduced by the air distribution and treatment system. However, bacterial concentrations and bioluminescence ATP values higher than the admissible values suggested by the relevant literature were found on about 30% of the total sampled surfaces. The ATP also documented the presence on some surfaces of a microbiological and biological contamination from the presence of traces of organic material and biological fluids.

During the various activities carried out in the autopsy room, the highest levels of airborne contaminants were found when the cranial cavity was opened. Other “critical” procedures with regard to potential exposure to pathogens were found to be those for opening the thoracic and abdominal cavities, since several pathogens belonging to Risk Group 2 were identified. The finding of similar biological agents requires the implementation of both collective and individual intervention measures aimed at protecting health in the workplace.

As far as endotoxins were concerned, the average value found in the course of autopsies after exhumation was approximately 33 times higher than that obtained in the course of autopsies carried out on corpses in a good state of conservation; the detection of an exposure peak during autopsies in exhumed corpses deserves great attention and prevention measures for risk containment. The concentrations of airborne bacteria (viable and cultivable), on the other hand, were superimposable. The greater presence of airborne endotoxins detected during the autopsy after exhumation could be due to the presence on the exhumed corpses of much higher concentrations of saprophytic bacteria than those of the mesophiles present on the bodies in good condition.

It is clear that the results of the study emphasized the need to provide for a more accurate cleaning and disinfection of surfaces, in particular, those on which high levels of contaminants were detected. It was also suggested to pay particular attention to the protection of the respiratory tract, especially when carrying out specific work procedures such as opening the cranial cavity and during autopsy investigations performed on exhumed corpses where operators were most exposed.

### 4.4. Research of SARS-CoV-2

Virological tests in the search for SARS-CoV-2 showed a significant rate of positivity among the bodies destined for autopsy, indicating a concrete risk of exposure for operators. The persistence of the viral genome beyond 10 days from death raises important reflections on the virulence of SARS-CoV-2 since RT-PCR was also able to detect viral RNA fragments deriving from post-mortem degradation.

In consideration of the above, regardless of the actual virulence and the history of no symptoms, it is advisable to adopt precautions for the prevention of infections by group 3 pathogens in cases of positive tests for SARS-CoV-2 [30,31,32,33].

## 5. Conclusions

Biorisk is an important element in the prevention of occupational accidents in forensic practice. Among all doctors, pathologists are considered to be at greatest risk from high blood exposure and the fact that judicial autopsies are performed on subjects with higher infectious risk for HBV, HCV and HIV [34,35].

The necropsy and morgue activity is characterized by the significant heterogeneity of tasks and related exposures in terms of both times and doses. The consequent risk assessment, therefore, cannot ignore the careful and timely examination of the operating methods and related procedures carried out in each individual workplace as well as the economic burden deriving from healthcare-associated infections [36,37,38].

The present study, owing to its relevance from a scientific point of view and its innovativeness evidenced by the scarcity of in-depth studies and research, can have significant repercussions on the safety of health professionals, allowing targeted interventions on current prevention and prophylaxis procedures, development of good practices for the workplace, and implementation of training courses.

Among the possible preventive modalities in forensic pathology, the recourse to post-mortem imaging methods stands out [39,40,41,42,43,44]. In fact, the possibility of preliminarily recognizing macroscopic pathological pictures or foreign bodies makes it possible to significantly limit the use of incongruous maneuvers and exposure to pathogens.

The management of risks associated with occupational exposure must necessarily include the accurate identification of hazards, the assessment of exposure methods, the adoption of risk-limiting measures, as well as the implementation of operational procedures that define the precautions and protections to be adopted [45,46,47].

The results obtained certainly provide indications for the identification of operating methods capable of limiting the spread of pathogenic microorganisms in the work environment and minimizing the biological risk [48,49,50]. The performance of monitoring activities made it possible to characterize the different sources of risk, including new ones, to which the different operators are exposed [51]. The search for pathogens on autopsy material and the analysis of the risk of transmission made it possible to focus attention on viral agents, allowing us to formulate a recommendation on the need to adopt adequate preventive measures, especially in the current pandemic scenario.

## Figures and Tables

**Table 1:** Sampling sites.

Areas and Equipment Sampled
90 morgue refrigeration units (+5 °C)
UV lamps for room disinfection
Full-air ventilation system with emission filters capable of ensuring 12.5 changes/h
No. 3 autopsy tables complete with accessories
1 refrigerator (+4 °C) and 1 freezer (−20 °C)
Autoclave
Hood for histology complete with accessories
Vacuum tissue processing machine
Semi-automatic microtome and automatic colorator
Microscope

**Table 2:** Qualitative analysis for SARS-CoV-2 RNA detection.

B-βCoV (Target E Gene)	SARS-CoV-2 (Target S Gene)	Internal Control	Result Interpretation
+	+	+	Positive: B-βCoV (target E gene) and SARS-CoV-2 (target S gene) specific RNA detected
+	-	+	Positive: B-βCoV (target E gene) specific RNA detected
−	+	+	Positive: SARS-CoV-2 (target S gene) specific RNA detected
−	−	+	Negative: Neither B-βCoV (target E gene) nor SARS-CoV-2 (target S gene) specific RNA detected

**Table 3:** Search results for common bacteria and fungi.

Cases (No.)	Microorganisms	CFU
1	*Candida* spp. *Stenotrophomonas maltophilia*	>10^6^ CFU/mL >10^6^ CFU/mL
1	non-MDR *Klesbiella pneumoniae*	10^4^ CFU/mL
2	non-MDR *Klesbiella pneumoniae* *Candida* spp.	>10^6^ CFU/mL 10^2^ CFU/mL
1	Methicillin-susceptible *Staphylococcus aureus*	10^4^ CFU/mL
1	non-MDR *Klesbiella pneumoniae* Pseudomonas aeruginosa	10^4^ CFU/mL 10^4^ CFU/mL

MDR, Multi-Drug Resistant. CFU, Colony Forming Units.

**Table 4:** Pathogenic bacterial species isolated from air samples collected during the autopsies.

Activity	Bacteria
Skull opening	*Staphylococcus aureus*
Thoracic cavity opening	*Escherichia coli, Staphylococcus aureus, Enterococcus faecalis*
Abdominal cavity opening	*Escherichia coli, Enterobacter cloacae, Klebsiella oxytoca, Staphylococcus aureus, Enterococcus faecalis*

**Table 5:** Risk analysis of necropsy activities and impact of PPE in preventing risks.

Event	Related Activities	Bioaerosol Exposure Risk	Fluid Exposure Risk	Impact of PPE on Risk Prevention				
				Headgear	Visor or Glasses	Mask	Gown	Footwear
Acupuncture	Mobilization of the corpse, blood collection, tissue sampling	None	High	High	Low	Low	Low	Medium
Cut	Cavities opening, tissue sampling	None	High	High	Low	Low	Low	Medium
Jetting and leaking of biological fluid and organic material	External examination, cavities opening, tissue sampling, washing of stretchers	Low	High	High	Low	High	High	High
Sample droplets	Cavities opening, tissue sampling, washing of stretchers	High	Medium	Medium	Low	High	High	Low

## Data Availability

The data that support the findings of this study are available from the corresponding author upon reasonable request.
